# Automated recognition of spontaneous facial expression in individuals with autism spectrum disorder: parsing response variability

**DOI:** 10.1186/s13229-020-00327-4

**Published:** 2020-05-11

**Authors:** Abigail Bangerter, Meenakshi Chatterjee, Joseph Manfredonia, Nikolay V. Manyakov, Seth Ness, Matthew A. Boice, Andrew Skalkin, Matthew S. Goodwin, Geraldine Dawson, Robert Hendren, Bennett Leventhal, Frederick Shic, Gahan Pandina

**Affiliations:** 1 Neuroscience Therapeutic Area, Janssen Research & Development, Titusville, NJ USA; 2 Digital Phenotyping Group, Discovery Sciences, Janssen Research & Development, Spring House, PA USA; 3 Digital Phenotyping Group, Discovery Sciences, Janssen Research & Development, Beerse, Belgium; 4 Bouvé College of Health Sciences, Northeastern University, Boston, MA USA; 5 Department of Psychiatry and Behavioral Sciences, Duke Center for Autism and Brain Development, Duke Institute for Brain Sciences, Duke University, Durham, NC USA; 6 Department of Psychiatry, School of Medicine, University of California, San Francisco, San Francisco, CA USA; 7 Center for Child Health, Behavior and Development, Seattle Children’s Research Institute, Seattle, WA USA; 8 Department of Pediatrics, University of Washington, Seattle, WA USA

**Keywords:** Autism spectrum disorder, Emotions, Emotional regulation, Facial expression, Impulsive behavior

## Abstract

**Background:**

Reduction or differences in facial expression are a core diagnostic feature of autism spectrum disorder (ASD), yet evidence regarding the extent of this discrepancy is limited and inconsistent. Use of automated facial expression detection technology enables accurate and efficient tracking of facial expressions that has potential to identify individual response differences.

**Methods:**

Children and adults with ASD (*N* = 124) and typically developing (TD, *N* = 41) were shown short clips of “funny videos.” Using automated facial analysis software, we investigated differences between ASD and TD groups and within the ASD group in evidence of facial action unit (AU) activation related to the expression of positive facial expression, in particular, a smile.

**Results:**

Individuals with ASD on average showed less evidence of facial AUs (AU12, AU6) relating to positive facial expression, compared to the TD group (*p* < .05, *r* = − 0.17). Using Gaussian mixture model for clustering, we identified two distinct distributions within the ASD group, which were then compared to the TD group. One subgroup (*n* = 35), termed “over-responsive,” expressed more intense positive facial expressions in response to the videos than the TD group (*p* < .001, *r* = 0.31). The second subgroup (*n* = 89), (“under-responsive”), displayed fewer, less intense positive facial expressions in response to videos than the TD group (*p* < .001; *r* = − 0.36). The over-responsive subgroup differed from the under-responsive subgroup in age and caregiver-reported impulsivity (*p* < .05, *r* = 0.21). Reduced expression in the under-responsive, but not the over-responsive group, was related to caregiver-reported social withdrawal (*p* < .01, *r* = − 0.3).

**Limitations:**

This exploratory study does not account for multiple comparisons, and future work will have to ascertain the strength and reproducibility of all results. Reduced displays of positive facial expressions do not mean individuals with ASD do not experience positive emotions.

**Conclusions:**

Individuals with ASD differed from the TD group in their facial expressions of positive emotion in response to “funny videos.” Identification of subgroups based on response may help in parsing heterogeneity in ASD and enable targeting of treatment based on subtypes.

**Trial registration:**

ClinicalTrials.gov, NCT02299700. Registration date: November 24, 2014

## Introduction

Individuals with ASD show difficulties in reciprocal social interactions. Conveyance of emotional states through facial expression constitutes one facet of such interactions, and differences in use of facial expressions are a diagnostic feature of ASD [1]. However, current work examining facial expressivity in ASD is conflicted. In general, studies have shown that individuals with ASD display diminished (flat) or atypical responses [2–6], though there is also evidence that degree of expressiveness in ASD may be different, rather than impaired [7–9] with some individuals being equally, or more expressive than TD controls.

While some variability in findings across studies may be accounted for by differences in study design and measurement of facial expression, variability could also be due to the heterogeneity within ASD. For instance, when asked to display a specific emotional facial expression, individuals with ASD (age 6 years to adult) have been found to be generally less expressive than a comparison TD group. However, the response in the ASD group was highly variable, with some individuals demonstrating more intense or exaggerated expression than the TD group, and for a longer duration. Moreover, Trevisan et al. [10] found that positive or negative response to emotional videos did not differ between ASD (*n* = 17) and TD (*n* = 17) groups of children (aged 7–13 years); however, variability in response related to reported alexithymia (difficulty identifying and expressing emotions) did. In this case, those with ASD and alexithymia were less facially expressive in their response to videos.

It is possible that co-occurring conditions or additional factors influence individual differences in emotional expression within ASD [10–13]. For example, the capacity for emotional regulation (ER)—the process whereby an individual can appropriately increase, decrease, or sustain emotions—may be delayed or altered in ASD, and to differing degrees [14, 15]. Individual differences regulating affective experiences have been found to associate with variability in cognition, social processing, and brain functioning [16, 17]. Such differences could also be related to comorbid internalizing and externalizing disorders in ASD, which some have suggested may contribute to the development of psychiatric disorders in general [15, 18]. While individual differences in ER abilities are generally underrepresented in studies of ASD, Mazefksy has proposed that they are a key dimension by which individuals with ASD may vary [19]. Differences in suppression of emotional response may be a factor that explains inconsistent findings across ASD studies of emotional expression. If individuals with ASD do not modulate emotional responses, this may be because they do not interpret the social setting and understand the rules of social display or because difficulties with ER prevent them from doing so [9]. Understanding the heterogeneity of emotional response in ASD and how it relates to other phenotypic characteristics is important in planning and evaluation of intervention.

In comparison studies of TD and ASD facial expression, the limited sizes of ASD groups have impacted the ability to investigate and understand phenotypic differences that might lead to differences in facial expression. One bottleneck in facial expression studies is the rigorous manual coding of emotional expression through facial affect coding. However, the advent of new computer vision software capable of automated facial expression analysis and subsequent reductions in analysis time has enabled researchers to obtain larger samples of individuals with ASD [20]. For example, the Autism and Beyond study utilized automatic coding of over 4000 video samples to establish differences in emotion expression in toddlers who had a high likelihood of future ASD diagnosis [21]. Our group has also previously reported on the use of facial expression analysis software (FACET), an automatic facial recognition technology that can be used to obtain evidence of a particular emotion, using a combination of action units (AUs), or to indicate evidence of specific AUs in isolation. Action units are the individual or groups of muscle movements that make up the Facial Action Coding System [22].

The aim of the current study was to investigate spontaneous production of facial expressions associated with positive emotions in a large group of individuals with ASD. We use the term spontaneous to distinguish from other studies where facial responses are prompted, either verbally or with a visual prompt and request to mimic. The purpose was to identify a practical clinical response variable that might be useful in parsing heterogeneity and measuring response to intervention in ASD. Therefore, “funny video” clips were used, meaning that spontaneous responses to the same prompt could be measured across participants. Action units (AU) [23] (AU6 cheek raiser, AU12 lip corner puller) were compared between ASD and TD groups and within the ASD group. Both AU6 and AU12 [24] were used together to account for the differences observed between posed or non-Duchenne smile, which tends to be represented in a single action unit (AU12), and a Duchenne or real smile.

## Hypotheses

First, it was hypothesized that, as an entire group, individuals with ASD would have a less dynamic or intense spontaneous positive facial emotional response demonstrated by activation of AU12 and/or AU6 when viewing “funny videos” as compared with TD control participants, and that differences in facial emotional response in the ASD group might be related to caregiver-reported phenotypic characteristics.

Second, we hypothesized that variability in positive facial emotional response in the ASD group may lead to definable subgroups, with different patterns of spontaneous facial affect, compared to each other, and to the TD group. It was predicted that individuals with ASD who show a different response pattern may also differ in social communication skills and in other caregiver-reported behaviors related to ER.

## Methods

This study was part of a larger prospective, non-interventional, multicenter, clinical trial (NCT02299700) wherein TD and ASD participants viewed a variety of standardized stimuli while eye-tracking, electroencephalogram, facial expression, and physiological biosensor data were collected [25]. This study was conducted from 06 July 2015 to 14 October 2016 at 9 study sites in the US.

### Participants

#### ASD Sample

The study enrolled males and females aged ≥ 6 years with a confirmed diagnosis of ASD according to the Autism Diagnostic Observation Schedule, 2nd edition (ADOS-2) [26]. Key exclusion criteria were a measured composite score on the Kaufmann Brief Intelligence Test-2 (KBIT-2) [27] of < 60 during screening (or other recent intelligence quotient [IQ] evaluation). In addition, ASD participants with a history of or current significant medical illness, and psychological and/or emotional problems not associated with ASD that the investigator considered should exclude the individual, for example render the informed consent invalid or limit the ability of the individual to comply with the study requirements. The inclusion criteria for participants with ASD were that they had a caregiver who had regular contact with them and was filling in various questionnaire measures, including those used in this analysis.

#### Control Sample

Participants in the control sample were TD males and females, aged ≥ 6 years, with a score in the normal range on the Social Communication Questionnaire [28], who had no major mental health disorder per the *Diagnostic and Statistical Manual of Mental Disorders*, *4th/5th Edition* [1] per the MINI International Neuropsychiatric Interview-6.0 (MINI) or MINI International Neuropsychiatric Interview-6.0, Pediatric Component (MINI-KID), or significant medical illness, and were not taking psychotropic medication. This TD cohort provided normative data for comparison with that from participants with ASD participants. It was deliberately smaller, since the primary goal of the study was to investigate the practicality of obtaining quality biosensor data from individuals with ASD and to investigate the heterogeneity within this group.

## Materials

### Funny Videos Task

Videos were chosen from a library used in America’s Funniest Home Videos and licensed for use in this study (Cara Communications, Los Angeles, CA, USA). Selections were made based on responses of individuals in a previous, unpublished study. Ten videos indicating change in positive emotional responses in TD groups were initially selected for presentation in a pilot study, for example observation of smiling and laughing when watching the videos and verbal report that the videos were amusing. Three videos (each between 13 and 20 sec long) for use in this study were selected on the basis that they evoked some changes in emotional response (measured using FACET) in both ASD and TD groups during the pilot study [29].

Video 1, *5 start a flood*, showed a group of children playing and climbing on an inflatable pool that subsequently collapses and sends the group sliding down a grass bank. Video 2, *Dinosaur drop*, showed a birthday celebration where the dinosaur birthday cake accidentally drops on the floor, much to the amusement of the family. Video 3, *Car riding canine*, showed a dog enjoying a car ride with an open window, causing his jowls to vibrate and expose his teeth. Selection was influenced by ensuring variability in the clips, to include some that required a degree of mentalizing and others that were assumed to require less. In order to maximize the valid data and observe variability in responses, we combined the responses across 3 videos.

The videos were presented in the same order and position for all participants as part of a larger battery of tasks lasting approximately 30 min [25]. Participants sat in a comfortable chair approximately 60 cm from a 23-inch computer screen (1920 × 1080 pixels). The height of the chair and screen were adjusted to ensure that participants’ eyes were level with the center of the screen. Two study staff were present in the room, one behind the participant, with interaction for redirection only, and one behind a screen operating the stimulus presentation software. At the beginning of the study, participants were instructed to pay attention to the screen, with no other specific instruction. If their attention to the screen wavered, they were prompted and reminded to look at the screen, and breaks were given as required. Interaction between the participant and the experimenters was deliberately kept to a minimum.

### Scales

Parents or caregivers of individuals with ASD completed the following scales:

*Social Responsiveness Scale 2™* (*SRS-2*). The SRS-2 [30] identifies the presence and severity of social impairment in ASD. It contains 65 items intended to assess social communication and restricted and repetitive behaviors. The social communication domain of the SRS-2 was compared with facial expression results.

*Aberrant Behavior Checklist* (*ABC*). The ABC [31, 32] is a 58-item behavior rating scale used to measure behavior problems across five subscales: (1) Irritability, (2) Social Withdrawal, (3) Stereotypic Behavior, (4) Hyperactivity/Noncompliance, and (5) Inappropriate Speech. Based on our hypotheses, we selected Irritability, Social Withdrawal, and Hyperactivity domains of the ABC to compare with facial expression results.

*Autism Behavior Inventory* (*ABI*). The ABI [33, 34] consists of 73 items across the following 5 domains: (1) Social Communication, (2) Restrictive Behaviors (resistance to change, Stereotypical Behavior, and Hypersensitivity), (3) co-occurring symptom domains of Mood and Anxiety, (4) Self-regulation (inattentiveness, impulsiveness, overactivity, and sleep issues), and (5) Challenging Behavior. We selected ABI domains and subdomains associated with Self-regulation (impulsivity) and Hypersensitivity given these are behaviors that may be related to ER and not fully captured in the other scales used in this study.

### FACET

The FACET program is based on the Computer Expression Recognition Toolbox (CERT), a system for automatically coding 19 different Facial Action Codes as well as 6 different prototypical facial expressions plus neutral [35]. CERT achieves an accuracy of 90.1% on a database of posed facial expressions and nearly 80% on a spontaneous facial expression dataset. FACET calculates the AU activation relative to an “emotional baseline”—a measure of activation determined during a period of time before the experiment where no stimulus is present. FACET software requires that the participant’s eyes and face are detected; therefore, the expressions are analyzed only when the participant is facing the screen where the stimuli are displayed. This is not a guarantee of attention but the closest proximity available.

### Feature extraction

For each frame of the video, raw data is collected in the form of evidence values for activated AUs and estimated as ${\log}_{10}\frac{P\left(\mathrm{AU}\ \mathrm{is}\ \mathrm{active}\right|\mathrm{data}\Big)}{1-P\left(\mathrm{AU}\ \mathrm{is}\ \mathrm{active}\right|\mathrm{data}\Big)}$ . Here *P*(AU is active|data) is a posterior probability that the AU is active based on the information obtained from video data. For each AU, evidence values were extracted frame by frame over the duration of video, which were then aggregated to obtain and compare features within and across participant populations. For each video, the following features were extracted for each AU: Average AU evidence displayed over the duration of the video, and area under the absolute value of (AU Evidence) curve for the duration of the video (referred to as AUC). The average AU evidence feature was designed to capture the average reported value of evidence of emotion which includes valence (negativity or positivity on the scale) over the duration of video, while the absolute value of the area under the curve (AUC) intends to capture the strength or energy content of the signal, regardless of valence. These features, which were extracted from individual videos for a given AU, were then averaged across all three videos together to test hypotheses within this study.

The video recording was at a rate of 24 frames/sec, which generated anywhere from 936 to 1440 data points for each subject. Frank et al. [36] estimated that spontaneous smiles typically last 3–4 sec. The responses to 3 videos were combined to ensure that durations were adequate to capture the full duration of the spontaneous response while minimizing the risk of losing the subjects’ sustained attention for the full battery.

### Statistical analysis

All features extracted from each video were averaged over 3 videos, to derive a final set of 4 features that was then used for all subsequent analysis: average AU6, average AU12, and AUC AU6 and AU12. Differences in features for each AU between ASD (entire group and subgroups) and TD groups were assessed using a linear regression model controlling for sex and age:

$$ \mathrm{feature}\sim \mathrm{group}\ \left(\mathrm{TD}\ \mathrm{or}\ \mathrm{ASD}\right)+\mathrm{age}+\mathrm{sex} $$

Associations between AU features and other pre-defined caregiver-reported scales in the ASD entire group and subgroup were analyzed using partial Spearman correlation with sex, age, and IQ, as covariates:

$$ \mathrm{feature}\sim \mathrm{scale}+\mathrm{age}+\mathrm{sex}+\mathrm{IQ} $$

Group differences and correlation analysis have been done on ranks so that analysis is not affected by outliers in the data.

ASD subgroups were obtained by applying a Gaussian mixture model (GMM) to each participant’s average AU6 and average AU12 features. Since smiles can comprise both Duchenne smile (caused by the activation of AU6 and AU12) and non-Duchenne smile (caused by activation of AU12 only), we clustered ASD participants’ positive facial expressions by using both AU6 and AU12 features. To aid in developing a simpler interpretable model, we specifically performed the clustering using only average AU12 and average AU6 features. Further, since AUC AU evidence features capture the intensity of evidence of emotion, it is related to average AU evidence features. Therefore, using AUC AU12 and AUC AU6 features is expected to provide similar results of clustering as using Average AU12 and Average AU6 features, as also shown in Additional file Table 1. Moreover, visualization in two dimensions (average AU12 and average AU6 features) offered easier interpretation of clustering results than that in the case of four dimensions (average AU12, average AU6, AUC AU12, and AUC AU6). Thus, average AU12 and average AU6 features served as a good choice for clustering using the GMM. A detailed mathematical description of GMM is given below.

If ***x*** = {***x***_1_, ***x***_2_, …, ***x***_*i*_, …, ***x***_*n*_} is a set of *n* independent and identically distributed observations, then the probability of every observation can be specified through a finite mixture model of *G* number of components:

$$ f\left({\boldsymbol{x}}_i;\boldsymbol{\Psi} \right)=\sum \limits_{k=1}^G{\uppi}_{\mathrm{k}}{f}_k\left({\boldsymbol{x}}_i;{\boldsymbol{\theta}}_{\boldsymbol{k}}\right) $$

where **Ψ =** {π_1_, …, π_G − 1_, ***θ***_1_, …, ***θ***_*G*_ } are the parameters of the mixture model,

*f*_*k*_(***x***_*i*_; ***θ***_***k***_) is the *k*^th^ component density for observation ***x***_*i*_ with parameter vector ***θ***_***k***_,

{π_1_, …, π_*G* − 1_} are the mixing weights or probabilities; ${\uppi}_{\mathrm{k}}\ge 0,\sum \limits_{k=1}^G{\uppi}_{\mathrm{k}}=1$

Keeping *G* constant, estimation of mixture model parameters **Ψ** is performed via the expectation-maximization (EM) algorithm. Specifically, for a GMM, a Gaussian distribution for each component is assumed such that *f*_*k*_(***x***; ***θ***_***k***_)~*N*(***μ***_k_; **Σ**_k_). In the GMM approach to clustering, each component of the mixture density is usually associated with a group or cluster. The probability that an observation ***x***_*i*_ belongs to each cluster *k* can be calculated, and then the observation assigned to that cluster with the highest probability. The clusters are ellipsoidal and centered at the mean vector ***μ***_k_. Geometric characteristics of the cluster such as its volume, shape, and orientation are determined by the covariance matrix **Σ**_k_. Covariance matrix **Σ**_**k**_ can be parameterized by eigenvalue decomposition: ${\boldsymbol{\Sigma}}_{\mathrm{k}}={\uplambda}_k{\boldsymbol{D}}_k{\boldsymbol{A}}_k{\boldsymbol{D}}_k^T$. Here, λ_*k*_ is a scalar and controls the volume of ellipsoid, ***A***_*k*_ is a diagonal matrix controlling the shape of density contour where (***A***_*k*_) = 1, and ***D***_*k*_ is an orthogonal matrix that specifies the orientation of ellipsoid.

## Results

### Demographics

Table 1 provides demographic details for ASD (*N* = 124) and TD (*N* = 41) participants included in the statistical analysis. Mean (standard deviation [SD]) age (years) of TD and ASD participants were 16.27 (13.18) and 14.97 (8.19). Mean (SD) IQ composite score of the ASD group was 99.25 (19.25).

**Table 1:** Participant characteristics

	TD, *N* = 41	ASD, *N* = 124	ASD over-responsive, *n* = 35	ASD under-responsive, *n* = 89
Age (years), mean (SD)	16.27 (13.18)	14.97 (8.19)	12.03 (4.82)	16.12 (8.94)
Median (range)	11 (6–63)	13 (6–54)	11 (6–28)	13 (6–54)
Male, *n* (%)	27 (65.85)	93 (75)	26 (74.29)	67 (75.28)
KBIT-2 IQ composite score, mean (SD)	NA	99.25 (19.25)	105.43 (14.98)	96.82 (20.25)
ADOS-2 CSS total score, mean (SD)	NA	7.59 (1.74)	7.09 (1.77)	7.79 (1.70)
Average AU12, mean (SD)	0.64 (0.63)	0.37 (0.77)	1.08 (0.78)	0.09 (0.57)
Average AU6, mean (SD)	0.34 (0.55)	0.31 (0.57)	1.02 (0.63)	0.038 (0.15)
Area under the curve AU12, mean (SD)	13.42 (8.04)	12.99 (8.6)	20.7 (10.66)	9.96 (5.15)
Area under the curve AU6, mean (SD)	6.74 (8.24)	7.21 (8.68)	17.63 (10.41)	3.11 (1.8)

*ADOS-2 CSS* Autism Diagnostic Observation Schedule-2 Calibrated Severity Score, *ASD* autism spectrum disorder, *AU* action unit, *IQ* intelligence quotient, *KBIT-2* Kaufmann Brief Intelligence Test-2, *SD* standard deviation, *TD* typically developing

### Hypothesis 1a: Differences in Features of Facial Affect Response between TD and total ASD group

**Fig. 1:**
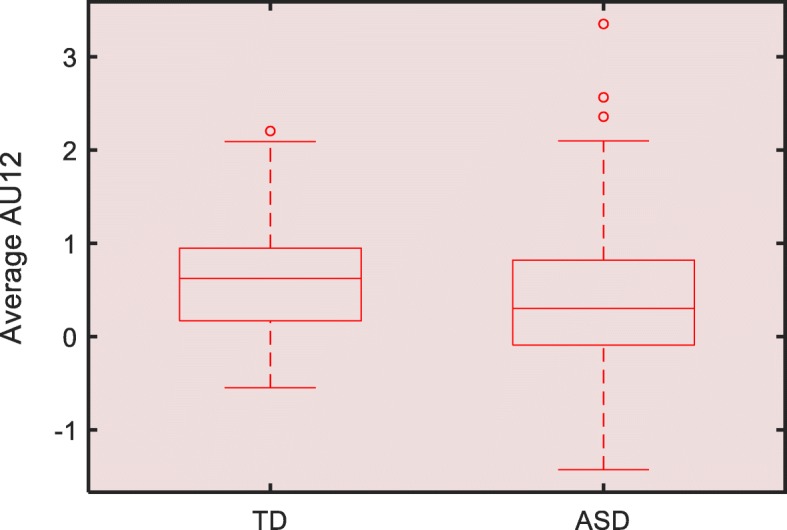
Plot of average AU12 between TD and ASD groups. ASD, autism spectrum disorder; AU, action unit; TD typically developing

We compared differences in features of each considered AU in response to funny videos between ASD and TD participants. As shown in Table 2, average AU12 was lower in the ASD group (*p* < .05). Figure 1 shows a box plot of average AU12 between ASD and TD groups. There were no other significant differences in FACET features observed between the ASD and TD group.

**Table 2:** AU6 and AU12 in response to funny videos

Features	ASD vs TD, *r* (*p* value)	95% CI for *r*
AU12 AUC	− 0.02 (.84)	[− 0.17, 0.14]
AU6 AUC	0.08 (.33)	[− 0.08, 0.23]
Average AU12	− 0.17 (.03)*	[− 0.32, − 0.02]
Average AU6	− 0.008 (.92)	[− 0.16, 0.15]

*ASD* autism spectrum disorder, *AU* action unit, *AUC* area under the curve, *TD* typically developing, *95% CI* 95% confidence interval

*p < .05

### Hypothesis 1b: Correlation of features of facial affect response with prespecified scales in the entire ASD group

Correlations between features for each AU and prespecified scales are shown in Table 3. ABI Hypersensitivity was significantly correlated with average AU12 (*p* < .05, *r* = 0.2). ABI Self-regulation–impulsivity was significantly correlated with AU12 AUC, AU6 AUC, and average AU6 (*p* < .01, *r* = 0.24; *p* < .05, *r* = 0.2; *p* < .05, *r* = 0.18, respectively).

**Table 3:** Correlations between features and scales in the entire ASD group

Scales	AU12 AUC	95% CI for *r*: AU12 AUC	AU6 AUC	95% CI for *r*: AU6 AUC	Average AU12	95% CI for *r*: Average AU12	Average AU6	95% CI for *r*: Average AU6
ABC-Hyperactivity Noncompliance	0.1 (0.24)	[− 0.07, 0.28]	− 0.001 (0.99)	[− 0.18, 0.17]	0.074 (0.42)	[− 0.1, 0.25]	0.05 (0.55)	[− 0.12, 0.23]
ABC-Irritability	0.005 (0.95)	[− 0.17, 0.18]	− 0.06 (0.49)	[− 0.24, 0.11]	0.075 (0.42)	[− 0.1, 0.25]	0.03 (0.73)	[− 0.15, 0.21]
ABC-Lethargy Social Withdrawal	− 0.09 (0.32)	[− 0.26, 0.09]	− 0.18 (0.05)	[− 0.34, − 0.002]	0.1 (0.25)	[− 0.07, 0.28]	− 0.002 (0.83)	[− 0.2, 0.16]
ABI RRB Hypersensitivity	0.05 (0.58)	[− 0.13, 0.22]	0.03 (0.75)	[− 0.15, 0.2]	0.2 (0.03)*	[0.02, 0.36]	0.07 (0.42)	[− 0.1, 0.25]
ABI Self-regulation	0.16 (0.09)	[− 0.02, 0.32]	0.07 (0.44)	[− 0.11, 0.24]	0.05 (0.59)	[− 0.13, 0.22]	0.05 (0.56)	[− 0.12, 0.23]
ABI SR Impulsivity	0.24 (0.009)*	[0.06, 0.4]	0.2 (0.03)*	[0.02, 0.36]	0.17 (0.12)	[− 0.05, 0.29]	0.18 (0.04)*	[0.003, 0.34]
RBS-R ritualistic behavior subscale total	0.14 (0.13)	[− 0.04, 0.31]	0.03 (0.71)	[− 0.14, 0.21]	0.29 (0.1)	[− 0.08, 0.27]	0.52 (0.06)	[− 0.12, 0.23]
SRS-2 Social Communication	− 0.11 (0.24)	[− 0.28, 0.07]	− 0.14 (0.13)	[− 0.31, 0.04]	− 0.001 (0.99)	[− 0.18, 0.18]	0.02 (0.82)	[− 0.16, 0.2]

Values are shown as *r* values (*p*)

*ABC* Aberrant Behavior Checklist, *ABI* Autism Behavior Inventory, *ASD* autism spectrum disorder, *AU* action unit, *AUC* area under the curve, *RBS-R* Repetitive Behavior Scale-Revised, *RRB* restrictive repetitive behaviors, *SRS-2* Social Responsiveness Scale 2, *SR* self-regulation, *95% CI* 95% confidence interval

*p < .05

### Hypothesis 2a: Gaussian mixture model approach to analyze expression of emotions in ASD

**Fig. 2:**
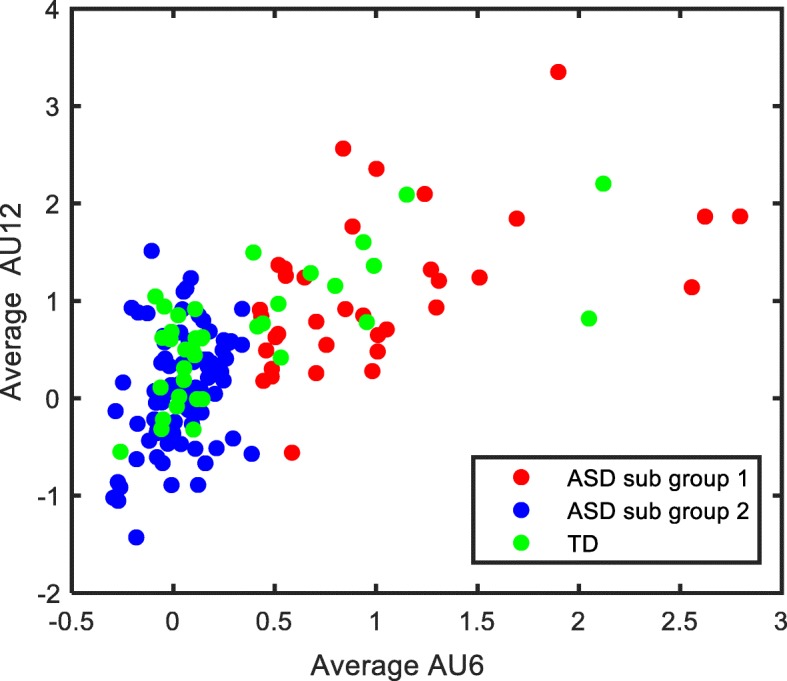
Scatterplot of average AU6 and average AU12. Red and blue dots represent average AU6 and average AU12 for over-responsive and under-responsive subgroups, respectively. The average AU6 and average AU12 values of participants from TD group are overlaid as green dots. ASD, autism spectrum disorder; AU, action unit; TD, typically developing

The ASD group exhibited large variability in average AU12 (mean = 0.37, SD = 0.77) compared to TD group (mean = 0.64, SD = 0.63). To parse the heterogeneity in the ASD group, we applied a GMM model on each ASD participant’s average AU12 and average AU6 to identify a cluster or subgroups of ASD. Features were normalized prior to cluster analysis. The number of clusters varied from 1 to 9, and a GMM model was implemented for each case. For a given prespecified number of clusters, the GMM model was then implemented for different geometric characteristics of clusters. In each case, the GMM model first calculated the probabilities that each observation (here an ASD participant) belonged to a certain cluster and then assigned an observation to the cluster with the highest probability. Cluster analysis was implemented in R software. Bayesian information criteria (BIC) were used to compare the performance of GMM model run on different number and geometry of clusters and is shown in Table 4A and Table 4B. The best performing model with BIC = 453.95 yielded 2 clusters or subgroups having variable volume, shape, and orientations. We termed one of these two subgroups as “over-responsive” (*n* = 35) and the other subgroup as “under-responsive” (*n* = 89). The former exhibited higher values of average AU6 and average AU12, while the latter subgroup exhibited lower values. Figure 2 shows a plot of average AU12 and average AU6 for two subgroups of ASD as identified by the model, overlaid with the corresponding values from TD group. Overlapping scores for FACET features and pre-specified scales for TD group and ASD subgroups are contained in the Additional file Tables 2, 3 and 4, Additional file Figures 1 and 2.

**Table 4:** Model BIC values for considering different number and geometric features of clusters

A														
Number of clusters	EII	VII	EEI	VEI	EVI	VVI	EEE	EVE	VEE	VVE	EEV	VEV	EVV	VVV
1	329.78	329.78	341.9	341.9	341.9	341.9	403.31	403.31	403.31	403.31	403.31	403.31	403.31	403.31
2	376.6	373.22	415.86	427.14	425.03	440.7	434.82	438.81	444.605	450.14	438.38	452.6205	437.93	453.95
3	406.28	411.32	413.85	429.21	440.04	440.02	421.63	443.24	434.301	411.5	450.51	436.6	442.15	434.24
4	404.88	433.3	443.2	433.52	428.52	430.11	441.53	427.6	431.14	433.36	438.25	430.14	425.19	418.89
5	398.21	426.21	440.49	427.24	411.01	408.63	427.07	423.25	440.24	414.86	431.7	414.98	422.75	400.51
6	389.66	425.29	426.22	417.25	391.91	401.25	412.56	420.74	424.87	395.56	416.85	398.13	405.2	394.02
7	376.13	410.3	411.71	395.8	392.15	386.09	406.97	417.57	400.4	384.73	409.35	385.69	394.08	369.96
8	393.65	403.24	403.08	390.8	389.57	373.56	414.45	413.31	394.81	375.29	404.46	378.46	384.64	354.54
9	386.29	386.79	392.99	386.61	373.08	352.27	402.13	395.38	381.66	358.28	390.7	357.03	371.26	328.17
B														
*Acronym of clusters and their corresponding geometric features are tabulated below:*														
Model			*Σ*_k_		Distribution			Volume			Shape			
EII			*λ****I***		Spherical			Equal			Equal			
VII			*λ*_*k*_***I***		Spherical			Variable			Equal			
EEI			*λ****A***		Diagonal			Equal			Equal			
VEI			*λ*_*k*_***A***		Diagonal			Variable			Equal			
EVI			*λ****A***_*k*_		Diagonal			Equal			Variable			
VVI			*λ*_*k*_***A***_*k*_		Diagonal			Variable			Variable			
EEE			*λ****DAD***^*⊤*^		Ellipsoidal			Equal			Equal			
EVE			*λ****DA***_*k*_***D***^*⊤*^		Ellipsoidal			Equal			Variable			
VEE			*λ*_*k*_***DAD***^*⊤*^		Ellipsoidal			Variable			Equal			
VVE			*λ*_*k*_***DA***_*k*_***D***^*⊤*^		Ellipsoidal			Variable			Variable			
EEV			*λ****D***_*k*_***AD***_*k*_^*⊤*^		Ellipsoidal			Equal			Equal			
VEV			*λ*_*k*_***D***_*k*_***A D***_***k***_^*⊤*^		Ellipsoidal			Variable			Equal			
EVV			*λ****D***_*k*_***A***_*k*_***D***_*k*_^*⊤*^		Ellipsoidal			Equal			Variable			
VVV			*λ*_*k*_***D***_*k*_***A***_*k*_***D***_*k*_^*⊤*^		Ellipsoidal			Variable			Variable

### Hypothesis 2b: Comparisons between ASD subgroups and TD group.

**Fig. 3:**
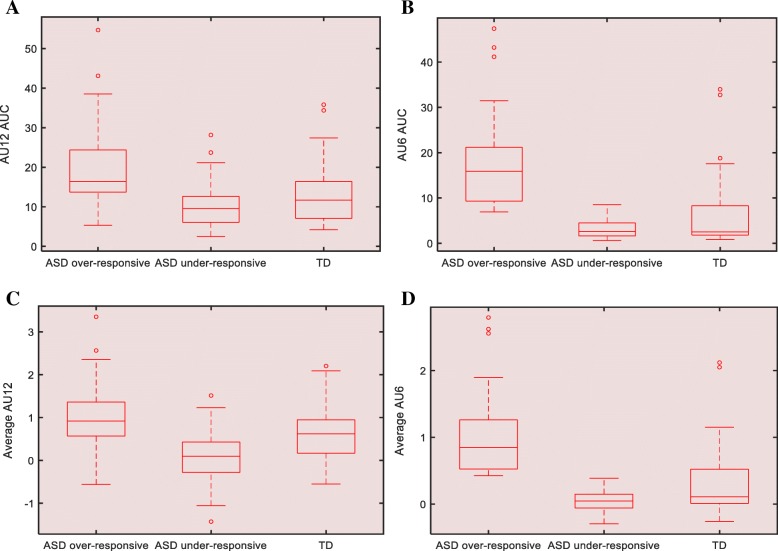
Plot of AU12 AUC (**a**), AU6 AUC (**b**), average AU12 (**c**), and average AU6 (**d**) between ASD subgroups and TD. ASD, autism spectrum disorder; AU, action unit; AUC, area under the curve; TD, typically developing

We also investigated differences in AU6 and average AU12 between participants in each ASD subgroup and TD group, using linear regression controlling for sex and age, given in Fig. 3. As shown in Table 5, both the average of AU6 and AU12 as well as AUCs of AU6 and AU12 in the over-responsive subgroup were significantly higher than the TD group (*p* < .001, *r* = 0.62 for average AU6; *p* < .001, *r* = 0.62 for AU6 AUC; *p* < .001, *r* = 0.31 for average AU12; *p* < .001, *r* = 0.41 for AU12 AUC). The under-responsive subgroup showed significantly lower values in average AU6 and average AU12 compared to the TD group (*p* < .001, *r* = − 0.25; *p* < .001, *r* = − 0.36, respectively). Mean (SD) IQ composite score of ASD subgroup 1, and ASD subgroup 2 were 105.43 (14.98), and 96.82 (20.25), respectively (Table 1).

**Table 5:** Difference in features between ASD subgroups and TD group

Features	ASD over-responsive vs TD	95% CI for *r*: ASD over-responsive vs TD	ASD under-responsive vs TD	95% CI for *r*: ASD under-responsive vs TD
AU12 AUC	0.41 (4e− 4)*	[0.19, 0.58 ]	− 0.16 (0.07)	[− 0.33, 0.01]
AU6 AUC	0.62 (7.7e− 9)*	[0.46, 0.75]	− 0.11 (0.22)	[− 0.28, 0.07]
Average AU12	0.31 (8.9e− 3)*	[0.09, 0.51]	− 0.36 (3.3e− 5)*	[− 0.51, − 0.2]
Average AU6	0.62 (1.2e− 8)*	[0.45, 0.74]	− 0.25 (6.1e− 3)*	[− 0.4, − 0.07]

Values are shown as *r* (*p* values)

*ASD* autism spectrum disorder, *AU* action unit, *TD* typically developing, *95% CI* 95% confidence interval

*p < .05

### Hypothesis 2c: Patterns of facial affect response within the ASD group.

#### Differences between ASD groups

**Fig. 4:**
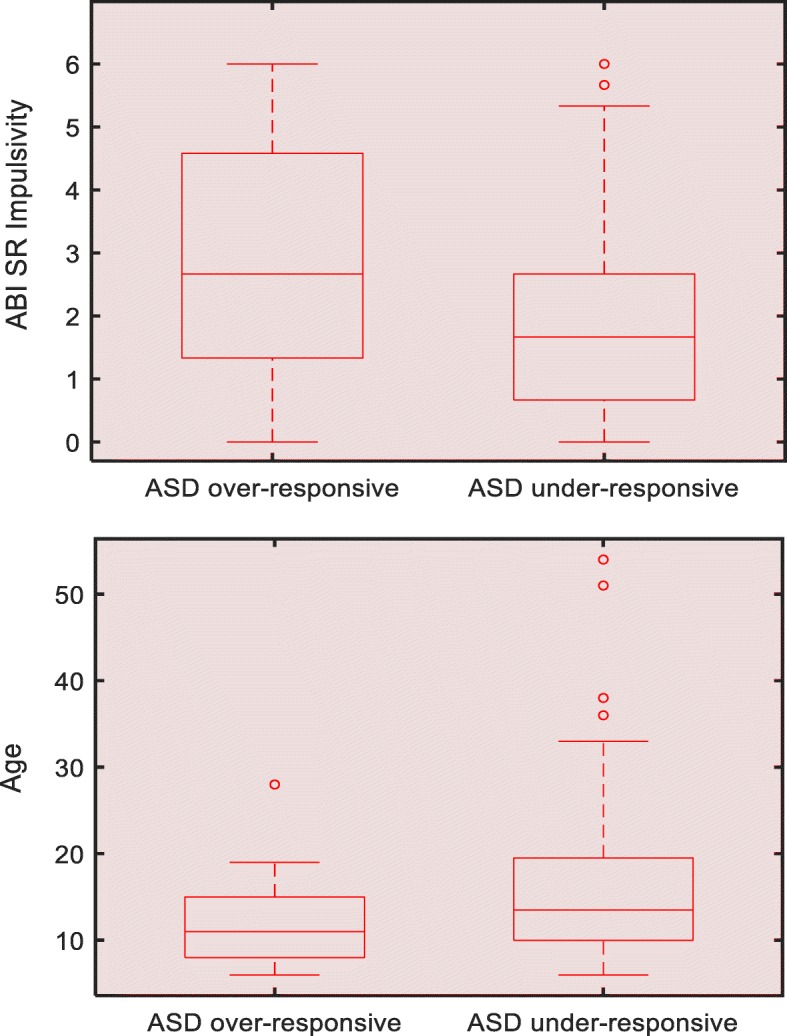
Plot of ABI SR Impulsivity (top) and age (bottom) between ASD subgroups. ASD, autism spectrum disorder; AU, action unit; AUC, area under the curve

Differences in features and pre-defined scales between the two ASD groups using linear regression controlling for age, sex, and IQ were assessed (Table 1). As shown in Table 6, the ABI Self-regulation–impulsivity scale showed a significant difference (*p* < .05, *r* = 0.21) between the two subgroups. All FACET features showed significant differences between the two subgroups (*p* < .001, *r* = 0.78 for average AU6; *p* < .001, *r* = 0.78 for AU6 AUC; *p* < .001, *r* = 0.55 for average AU12; *p* < .001, *r* = 0.51 for AU12 AUC). The over-responsive group was significantly younger than the under-responsive group (*p* < .05, *r* = − 0.21), as given in Fig. 4.

**Table 6:** Difference in scales, features, ADOS-2 CSS total, sex, age, and IQ between ASD subgroups

ASD over-responsive vs under-responsive		*r* (*p* value)	95% CI for *r*
Scales	ABC-Hyperactivity Noncompliance	0.06 (0.5)	[− 0.12, 0.23]
	ABC-Irritability	0.02 (0.86)	[− 0.16, 0.19]
	ABC-Lethargy Social Withdrawal	− 0.02 (0.86)	[− 0.19, 0.16]
	ABI RRB Hypersensitivity	0.15 (0.11)	[− 0.03, 0.31]
	ABI Self-Regulation	0.07 (0.46)	[− 0.11, 0.24]
	ABI SR Impulsivity	0.21 (0.02)*	[0.04, 0.38]
	RBS-R Ritualistic Behavior Subscale Total	0.15 (0.11)	[− 0.03, 0.31]
	SRS-2 Social Communication	− 0.03 (0.76)	[− 0.2, 0.15]
Features	AU12 AUC	0.51 (1.9e− 9)	[0.37, 0.63]
	AU6 AUC	0.78 (3.7e− 26)*	[0.7, 0.84]
	Average AU12	0.55 (1e− 10)*	[0.41, 0.66]
	Average AU6	0.78 (4.5e− 26)*	[0.7, 0.84]
Other	ADOS-2 CSS Total	− 0.15 (0.09)	[− 0.32, 0.02]
	Sex	− 0.05 (0.56)	[− 0.23, 0.12]
	Age	− 0.21 (0.02)*	[− 0.38, − 0.04]
	IQ	0.16 (0.06)	[− 0.009, 0.33]

*ABC* Aberrant Behavior Checklist, *ABI* Autism Behavior Inventory, *ADOS-2 CSS* Autism Diagnostic Observation Schedule-2 Calibrated Severity Score, *ASD* autism spectrum, disorder, *AU* action unit, *AUC* area under the curve, *IQ* intelligence quotient; *RBS-R* Repetitive Behavior Scale-Revised, *RRB* restrictive repetitive behaviors, *SRS-2* Social Responsiveness Scale 2, *SR* self-regulation, *TD* typically developing, *95% CI* 95% confidence interval

*p < .05

There were no significant differences between groups for ABI Mental Health Measures (Additional file Table 5).

#### Correlation of features within ASD subgroups

Controlling for age, sex, and IQ, we assessed associations between AU features and scales in the two ASD subgroups using partial Spearman correlation. No significant associations were obtained in the over-responsive group (Table 7). In the under-responsive group, the ABC-lethargy social withdrawal scale was significantly correlated with AU6 AUC (*p* < .01, *r* = − 0.3).

**Table 7:** Association between features and scales in ASD over-responsive (A) and under-responsive (B)

Scales	AU12 AUC	95% CI for *r*: AU12 AUC	AU6 AUC	95% CI for *r*: AU6 AUC	Average AU12	95% CI for *r*: Average AU12	Average AU6	95% CI for *r*: Average AU6
A								
ABC-Hyperactivity Noncompliance	0.11 (0.54)	[− 0.23, 0.43]	0.19 (0.29)	[− 0.15, 0.49]	0.15 (0.42)	[− 0.2, 0.46]	0.19 (0.29)	[− 0.15, 0.49]
ABC-Irritability	− 0.04 (0.82)	[− 0.37, 0.29]	0.08 (0.67)	[− 0.26, 0.4]	− 0.04 (0.84)	[− 0.37, 0.3]	0.08 (0.66)	[− 0.26, 0.4]
ABC-Lethargy Social Withdrawal	0.13 (0.46)	[− 0.21, 0.45]	− 0.05 (0.78)	[− 0.38, 0.29]	0.15 (0.41)	[− 0.19, 0.46]	− 0.07 (0.71)	[− 0.39, 0.27]
ABI RRB Hypersensitivity	0.02 (0.9)	[− 0.31, 0.35]	− 0.22 (0.23)	[− 0.51, 0.12]	0.06 (0.73)	[− 0.28, 0.39]	− 0.2 (0.27)	[− 0.5, 0.14]
ABI Self-Regulation	0.22 (0.23)	[− 0.12, 0.51]	0.23 (0.21)	[− 0.11, 0.52]	0.22 (0.22)	[− 0.12, 0.52]	0.2 (0.28)	[− 0.15, 0.5]
ABI SR Impulsivity	0.19 (0.29)	[− 0.15, 0.5]	0.34 (0.06)	[0.009, 0.61]	0.19 (0.29)	[− 0.15, 0.49]	0.28 (0.12)	[0.06, 0.56]
RBS-R Ritualistic Behavior Subscale Total	0.1 (0.6)	[− 0.25, 0.42]	− 0.19 (0.31)	[− 0.49, 0.16]	0.07 (0.7)	[− 0.27, 0.39]	− 0.22 (0.22)	[− 0.52, 0.12]
SRS Social Communication	− 0.14 (0.45)	[− 0.45, 0.2]	0.0007 (0.1)	[− 0.33, 0.33]	− 0.18 (0.33)	[− 0.48, 0.17]	− 0.025 (0.89)	[− 0.36, 0.31]
B								
ABC-Hyperactivity Noncompliance	0.02 (0.86)	[− 0.19, 0.23]	− 0.14 (0.19)	[− 0.34, 0.07]	0.03 (0.77)	[− 0.18, 0.24]	− 0.009 (0.93)	[− 0.22, 0.2]
ABC-Irritability	− 0.01 (0.93)	[− 0.22, 0.2]	− 0.17 (0.12)	[− 0.36, 0.04]	0.11 (0.3)	[− 0.1, 0.31]	0.02 (0.87)	[− 0.19, 0.22]
ABC-Lethargy Social Withdrawal	− 0.17 (0.11)	[− 0.37, 0.04]	− 0.3 (0.004)*	[− 0.48, − 0.1]	0.17 (0.12)	[− 0.04, 0.36]	0.03 (0.77)	[− 0.18, 0.24]
ABI RRB Hypersensitivity	− 0.03 (0.77)	[− 0.24, 0.18]	− 0.09 (0.43)	[− 0.29, 0.12]	0.19 (0.07)	[− 0.01, 0.39]	− 0.04 (0.72)	[− 0.25, 0.17]
ABI Self-Regulation	0.07 (0.54)	[− 0.14, 0.27]	− 0.02 (0.89)	[− 0.22, 0.19]	− 0.04 (0.72)	[− 0.24, 0.17]	− 0.03 (0.76)	[− 0.24, 0.18]
ABI SR Impulsivity	0.05 (0.64)	[− 0.16, 0.26]	− 0.02 (0.88)	[− 0.22, 0.19]	− 0.03 (0.75)	[− 0.24, 0.18]	− 0.009 (0.94)	[− 0.22, 0.2]
RBS-R Ritualistic Behavior Subscale Total	0.05 (0.66)	[− 0.16, 0.25]	− 0.13 (0.25)	[− 0.33, 0.08]	0.03 (0.79)	[− 0.18, 0.24]	− 0.07 (0.55)	[− 0.27, 0.14]
SRS Social Communication	− 0.08 (0.46)	[− 0.29, 0.13]	− 0.21 (0.05)	[− 0.4, 0.0003]	0.11 (0.33)	[− 0.1, 0.31]	0.09 (0.42)	[− 0.12, 0.29]

Values are shown as *r* (*p* values)

*ABC* Aberrant Behavior Checklist, *ABI* Autism Behavior Inventory, *AU* action unit, *RBS-R* Repetitive Behavior Scale-Revised, *RRB* restrictive repetitive behaviors, *SRS-2* Social Responsiveness Scale 2, *SR* Self-regulation, *95% CI* 95% confidence interval

*p < .05

## Discussion

The aim of this study was to compare facial expression response to funny videos of individuals with ASD compared to a TD group, with a view to identifying useful clinical response variable for diagnosis or parsing heterogeneity. As predicted, the ASD group demonstrated an overall reduced positive facial expression to funny videos, as determined by a significant reduction in average AU12 (upturned mouth corner) compared to the TD group. However, the effect size was small, and differences were not seen in AU6 (cheek raiser), or either of the AUC feature measures. For the total ASD group, there were positive correlations between Hypersensitivity and Self-regulation–impulsivity reports on the ABI and the FACET features, indicating that those individuals with ASD who displayed increased positive emotional response to the videos were reported by caregivers to be more hypersensitive and more impulsive.

Also, as predicted, we observed large AU variability in response to videos within the ASD group compared to the TD group, indicating that reduced evidence of facial emotional expression in response to the videos was *not* universal in the ASD group. Using a combination of average AU12 and AU6 in a Gaussian mixture model, we identified two subgroups of ASD responders—described as over-responsive and under-responsive. These subgroups differed significantly from each other on all four FACET features included in the statistical analysis. In addition, those in the over-responsive group were significantly more responsive, and those in the under-responsive group were significantly less responsive, than the TD group, respectively. This indicates that the under-responder group—represented by the majority of ASD participants in this study—responded in a way consistent with literature and with what might be expected based on the diagnostic criteria; however, a smaller subgroup—the over-responders—had a different response pattern. These differences would not have been accounted for by solely looking at mean differences between groups, or even by comparing group variability.

Comparison of the over- and under-responsive subgroups on core ASD features (ADOS-2; SRS-2 social communication) did not reveal any significant differences. The over-responsive subgroup was younger but did not differ significantly in terms of IQ. We compared the subgroups on scales that may associate with regulation of emotions and found that the over-responsive group was reported as significantly more impulsive than the under-responsive group. In examining whether different relationships between behavioral features and emotional responsiveness existed for the over-responsive and under-responsive subgroups, relationships between behavioral features and patterns of facial expression were found in the groups that had not been evident for the entire group. Lack of correlation for the overall group could be explained by the difference in expression of AU6 (Additional file Figure 3). Less expression of AU6 was associated with increased social withdrawal for the under-responsive group. By examining these groups separately, it may be possible to better understand some of the factors that might impact or result from affective over or under-responsiveness in the ASD group. For example, reduced facial emotional responsiveness could contribute to reported deficits in social interaction, specifically social withdrawal. In contrast, the over-responsive group was found to have more difficulties with impulsivity and control of their response, that could affect the ability to modulate emotional responses, or lack of emotional gating, and thus display more positive emotional expression than might typically be expected in response to the video. This increased affective response may be interpreted—either correctly or incorrectly—as hyperexcitability.

The relationship between increased expression of positive facial expressions in response to the videos with caregiver-reported impulsivity found in the over-responsive subgroup could be related to difficulties with emotional regulation, an under-studied area in ASD. Impairments in emotional regulation may lead to poorer behavior and outcomes for individuals with ASD [27, 28]. For instance, Zane et al. proposed that TD responses to similar funny videos, shown in an experimental setting, were socially modulated and governed by display rules, for example what is expected in a study setting with an unfamiliar experimenter [7]. Capps et al. also suggested that individuals with ASD are less likely to suppress or modulate responses according to rules of display, based on their study of emotional expression in toddlers with ASD [29]. There is debate as to whether emotional regulation difficulties are a part of the core features of ASD, or simply co-occur with other ASD symptoms [15]. Our results suggest that there may be a subgroup of individuals with ASD who show but do not modulate their emotional facial expressions, suggesting that they have more difficulty regulating their emotions than the TD group. This subgroup is reported to be more impulsive than other individuals with ASD who do not show the same response pattern. This impulsivity may relate to broader emotion regulation difficulties or affective lability that are evident in this subgroup. Better characterization of emotion regulation features in ASD is needed to draw more specific conclusions, and in the future, we would include a specific test of Emotional Regulation for comparison. In contrast, there is another group of individuals with ASD who demonstrate less facial emotional response to “funny videos” in the experimental setting. Different mechanisms may be driving this group’s reduced positive facial affect, such as alexithymia, reduced social interest or attention, or general reduced facial expressions in this group. The observation of relationship between social withdrawal symptoms in this group and not in the over-responsive group supports the theory that different mechanisms may be at play.

In addition, it may be useful to look at absolute differences in facial expression or action units when there is no stimulus present and to identify whether the subgroups differ in their facial appearance and expressivity in the absence of the response to the funny videos.

### Limitations

There are a number of limitations to our findings. Firstly, as this was foundational, exploratory work, our results do not account for multiple comparisons. However, the hypotheses were prespecified for between-group differences and associations with phenotype in the total group comparison, and we note that 6 out of 8 differences observed between over- and under-responsive ASD groups and the TD groups and the observation that less expression of AU6 was associated with increased social withdrawal for the under-responsive group would still remain significant with Bonferroni correction. Nevertheless, future work will have to ascertain the strength and reproducibility of all results. In addition, we have a large group of ASD individuals in our sample, with the deliberate purpose of understanding variability within each group. This resulted in an unequal sample size in ASD and TD groups which might bias the range of display of AUs associated with happiness in the ASD group, and there is also a lack of characterization of, in particular, IQ for the TD group, which also prevents from understanding differences in the relationships between cognitive functioning and emotional expression in this group. However, we do note in relation to IQ that there were no significant differences in IQ between ASD over-responsive and under-responsive groups (*p* > 0.05) (Table 5). If the observed differences in FACET features between ASD subgroup and the TD group were solely driven by differences in IQ, then we would have expected a comparable value of *r* (magnitude of between-group differences) between each ASD subgroup and TD group. Finally, there are other methodological considerations that might be accounted for in future research. For example, reduced facial expression does not necessarily equate to a lack of emotional arousal or reduced experience of happiness (we did not rate emotional arousal or internal states of emotion). Emotion regulation ability is composed of both affective experience and affective control [30]. We do not know which of these may have led to differences in facial expressiveness observed in our sample. It is difficult to assess the impact of the social setting on both ASD and TD participants. For example, there is a difference between facial expressions that have communicative intent in everyday social interactions, and facial expressions that are produced in response to funny videos under an explicit instruction from an experimenter and the participants noticed that they were being recorded. Some individuals may inhibit their responses due to perceived conventions and norms of what they might see as appropriate behavior in the study room. It is also not known whether there are any differences between groups in attempts to share laughter and amusement with the experimenter in the room. The social motivation theory of autism [31] would suggest that individuals with ASD differ more in what they smile/laugh at rather than how much, and there are many factors, both intrinsic and extrinsic, that may ultimately contribute to the expression of emotion in both TD and ASD individuals. In relation to this, it would be interesting to collect data regarding Alexithymia, the difficulty identifying and expressing emotions, and determine whether this measure differed between the subgroups identified.

### Clinical implications

The use of automatic facial recognition software enabled us to obtain data on facial affect expression from a larger than usual group of participants with ASD in an unobtrusive, accurate, and efficient way. Further, it allowed us to identify clusters or subgroups within the ASD group who differ significantly from each other and a TD group in response to funny videos. These findings support the notion that differences in facial expressions are evident in individuals with ASD. They also suggest that in order to understand the differences, we need to move beyond consideration of mean group differences and explore the existence of subgroups. Identifying subgroups within ASD may help explain some of the conflicting findings present in previous studies (e.g., Zane et al.) [7] that may display behavioral differences that are independent of severity of diagnosis. It may also provide a standardized and high-throughput way to parse some of the heterogeneity within ASD and enhance understanding of the complex relationship between differences in these subgroups and caregiver-reported observations. Our results support the notion of multiple dimensions of observable behavior that contribute to the autism phenotype [37], and the need to look at behaviors that go beyond the diagnostic criterion to consider profiles of skills across dimensions [38]. This could lead to personalization of interventions and an increased ability to link casual pathways to ASD phenotypes [39].

## Conclusions

We identified significant differences both between ASD and TD groups and within the ASD group who were either “over-responsive” or “under-responsive” to funny videos. Variability in facial expression response was associated with caregiver-reported impulsivity and may be related to emotional regulation. A relationship to caregiver-reported symptoms (social withdrawal) was found in the under-responsive group, but not the over-responsive group. These differences both between ASD and TD groups and within the ASD group suggests the potential utility of using automated facial expression response to naturalistic “funny videos” as a practical clinical response variable that might be useful in parsing heterogeneity and measuring treatment response in ASD.

## Supplementary information

**Additional file 1:.** Supplementary Material

## Data Availability

The data sharing policy of Janssen Pharmaceutical Companies of Johnson & Johnson is available at https://www.janssen.com/clinical-trials/transparency. As noted on this site, requests for access to the study data can be submitted through the Yale Open Data Access (YODA) Project site at http://yoda.yale.edu.

## Abbreviations

ABC: Aberrant Behavior Checklist
ABI: Autism Behavior Inventory
ADOS-2: Autism Diagnostic Observation Schedule, 2nd edition
ASD: Autism spectrum disorder
AU: Action units
AUC: Area under the AU Evidence curve
BIC: Bayesian information criterion
ER: Emotional regulation
FACET: Facial expression analysis software
KBIT-2: Kaufmann Brief Intelligence Test-2
IQ: Intelligence quotient
SRS-2: Social Responsiveness Scale 2™
SD: Standard deviation
TD: Typically developing
