# Supplementary material for: Automated recognition of spontaneous facial expression in individuals with autism spectrum disorder: parsing response variability
Source: Mol Autism. 2020 May 11;11:31. doi: 10.1186/s13229-020-00327-4 (PMC7212683; doi:10.1186/s13229-020-00327-4)
Supplement: Supplementary file 1 — Additional file 1:. Supplementary Material [file 13229_2020_327_MOESM1_ESM.docx]

**Supplementary Material**

**Choice of FACET features for clustering**

On application of the Gaussian Mixture Model (GMM) using only AUC AU12 and AUC AU6, the AU features in the two ASD subgroups showed no significant differences in mean (p>0.05) compared to corresponding features obtained from the original GMM model (which used average AU12 and average AU6 features for clustering). Table 1 shows the mean and standard deviation of all AU features for ASD over-responsive and under-responsive groups, when applying a GMM model on AUC AU12 and AUC AU6.


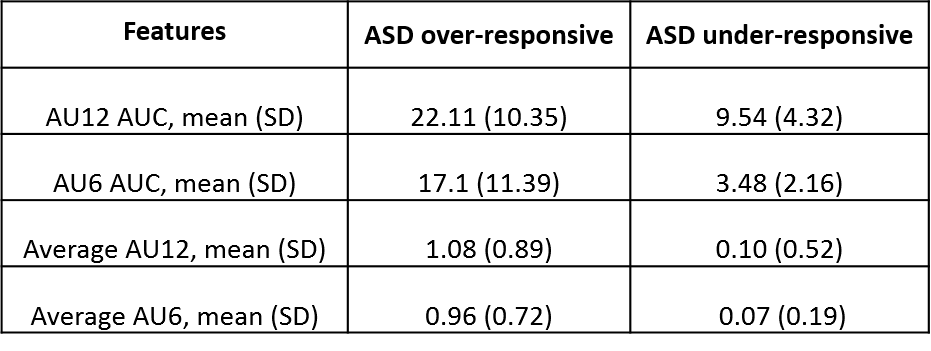


*Supplementary Table 1. Mean and standard deviation of features in ASD subgroups on application of a GMM model using AUC AU12 and AUC AU6 for clustering.*

**FACET Features: TD and ASD Overlapping Scores**

The distribution of each facet feature (AU12 AUC, AU6 AUC, Average AU12, Average AU6) for each group (TD group, ASD over-responsive subgroup, ASD under-responsive subgroup) was first fit to a normal distribution. For example, Figure 1 visualizes the distribution of Average AU12 in TD group overlaid on the distribution of Average AU12 in each ASD subgroup. Here we quantified the percentage overlap of a facet feature between an ASD subgroup and TD, by calculating the area of the overlap between the two corresponding distributions and normalizing it to the overall area [1]. Table 2 shows the overlap in values of facet features between TD group and each ASD subgroup.


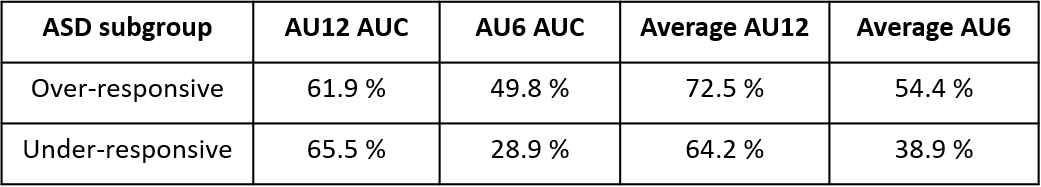


*Supplementary Table 2. Percentage overlap in facet features between TD and each ASD subgroup.*

A

B

*Supplementary Figure 1: Distribution of Average AU12 in TD group overlaid on the distribution of AU12 in ASD over-responsive (A) and ASD under-responsive (B) subgroups. Solid lines represent fit to a normal distribution.*

**Caregiver Reported Scales: TD and ASD Overlapping Scores**

Out of all caregiver reported scales reported in this manuscript, measurements of ABI RRB Hypersensitivity, ABI Self-regulation and ABI SR Impulsivity were available for individuals in the TD group. We calculated the range of values for each of the scales in the TD group and quantified the extent to which it overlapped with the corresponding range of values of scales in each ASD subgroup. Table 3 below shows the overlap in values of scales between TD group and each ASD subgroup.


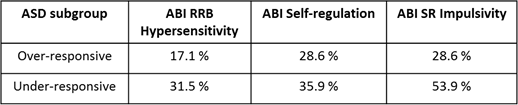


*Supplementary Table 3. Percentage overlap in values of caregiver reported scales between TD and each ASD subgroup.*

Figure 2 provides a visualization of the distribution of values of scales for each ASD subgroup. The shaded grey bars overlaid on each distribution, indicate the minimum and maximum values of the corresponding scales in the TD group.


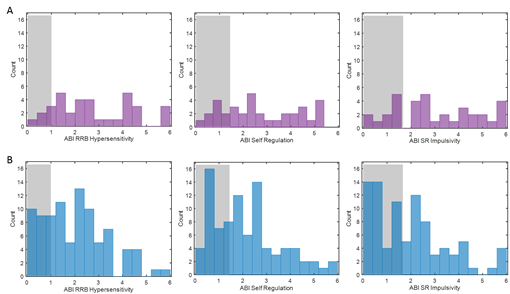


*Supplementary Figure 2. Distribution of scales in ASD over-responsive group (A) and ASD under-responsive group (B). Grey bars denote minimum and maximum values of scales in TD group.*

We further assessed for difference in scales between TD group and each ASD subgroup using a linear regression model controlled for age and sex. As shown in Table 4 below, significant differences in caregiver reported scales (p < 0.05) were observed between TD group and each ASD subgroup.


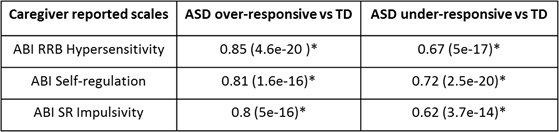


*Supplementary Table 4. Differences in scales between TD group and ASD subgroups. Values are shown as r (p-values).*

**Mental Health Measures**

Caregivers of individuals with ASD completed the *Autism Behavior Inventory Mental Health Anxiety* (ABI MH Anxiety) and *Autism Behavior Inventory Mental Health Depression* (ABI MH Depression), subsets of the *ABI* and use items related to anxiety and depression (Bangerter et al, 2017). Mean (SD) of the ABI MH Anxiety for entire ASD group, ASD over-responsive group, and ASD under-responsive group were 1.91 (1.23), 1.77 (1.15) and 1.97 (1.27) respectively. Mean (SD) of the ABI MH Depression for entire ASD group, ASD over-responsive group, and ASD under-responsive group were 1.45 (1.43), 1.43 (1.4) and 1.46 (1.45) respectively.

We assessed for the difference in each scale (ABI MH Anxiety and ABI MH Depression) between the two ASD subgroups using a linear regression model controlled for sex, age and IQ, but found no significant difference (p > 0.05 for each scale). We also assessed associations between each facet feature (AU12 AUC, AU6 AUC, Average AU12, Average AU6) and each of the two scales, in the ASD entire group and subgroups, using partial Spearman correlation with sex, age and IQ as covariates. As shown in Table 5, neither the ASD entire group nor the ASD subgroups showed any significant correlation (p> 0.05) between any FACET feature and any scale.


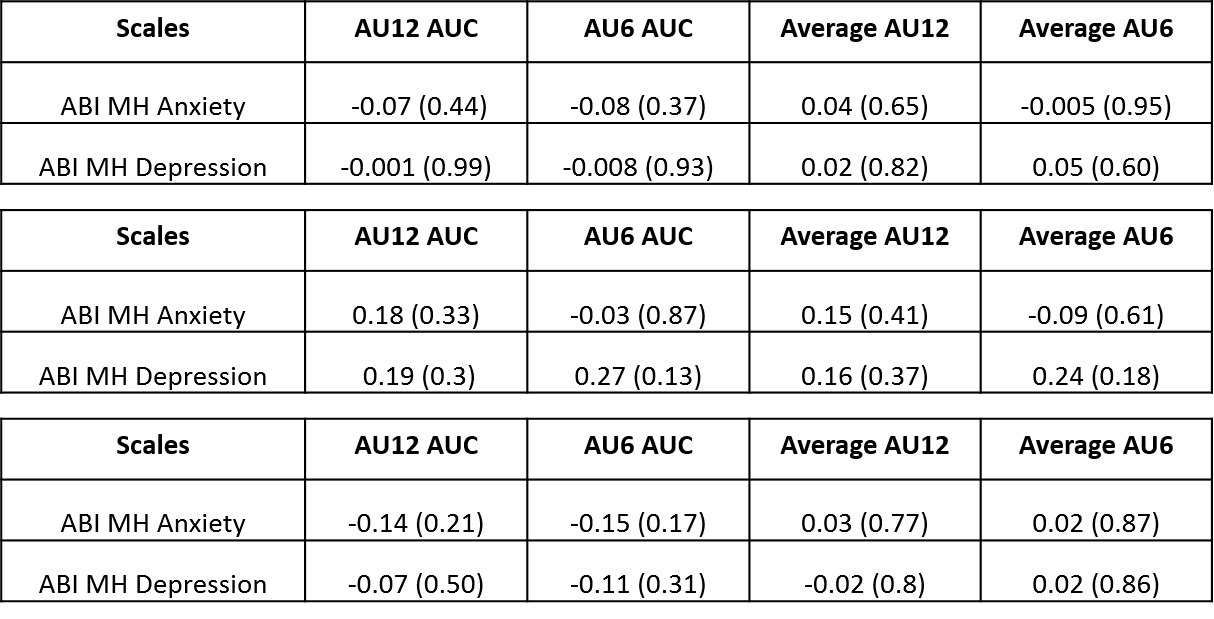


*Supplementary Table 5. Correlation between features and scales in entire ASD group (top), ASD over-responsive group (middle) and ASD under-responsive group (bottom). Values are shown as r (p-values).*

**Correlations Within ASD Groups**

The under-responsive group showed a negative correlation between social withdrawal symptoms and AU6 AUC- such that individuals with ASD who had lower AU6 AUC also had a higher value of ABC-Lethargy Social Withdrawal Scale. On collapsing the two subgroups, there was no evidence of significant relationship in the entire ASD group as the over-responsive group had no associations between AU6 AUC and ABC-Lethargy Social Withdrawal Scale. As an example, Figure 3 shows a plot of AU6 AUC and ABC-Lethargy Social Withdrawal Scale, on which a (linear) best-fit line for each ASD subgroup is overlaid as a guide to the eye. The slope of the best-fit line in ASD under-responsive group was -1.79 while that of the ASD over-responsive group was only 0.04 (slope corresponding to entire group was 0.06). This suggests that the lack of correlation for the overall group could be explained by the difference in expression of AU6 between the over-responders and the under-responders.

*Supplementary Figure 3. Plot of AU6 AUC and social withdrawal symptoms in each ASD subgroup. Colored solid lines represent best linear fit for each ASD subgroup.*

**References**

1. Henry F. Inman, Edwin L. Bradley Jr. The overlapping coefficient as a measure of agreement between probability distributions and point estimation of the overlap of two normal densities. Communications in Statistics - Theory and Methods. 1989; 18(10), 3851-3874.
